# Native liver survival and genetic associations in Korean patients with Alagille syndrome

**DOI:** 10.1007/s00431-026-06917-3

**Published:** 2026-04-10

**Authors:** Shinjie Choi, Dong-Uk Kim, Yeji Kim, Lia Kim, Jung Ok Shim, Jin Soo Moon, Jong Woo Hahn, Hye Ran Yang, Ju Young Chang, Jae Sung Ko

**Affiliations:** 1 Department of Pediatrics, Seoul National University College of Medicine, Seoul, Korea; 2 Department of Pediatrics, Seoul National University Hospital, Seoul, Korea; 3 Department of Pediatrics, Seoul National University Bundang Hospital, Seongnam, Korea; 4 Department of Pediatrics, Seoul Metropolitan Government-Seoul National University Boramae Medical Center, Seoul, Korea

**Keywords:** Alagille syndrome, Genetic variation, Liver transplantation, JAG1 protein, human, Receptor, NOTCH2

## Abstract

Alagille syndrome (ALGS) is a rare multisystem disorder most commonly resulting from pathogenic variants in *JAG1* and, less frequently, *NOTCH2*. We evaluated long-term native liver survival (NLS) and overall survival (OS) in a Korean ALGS cohort and compared the genetic characteristics of this cohort with those of the Global ALagille Alliance (GALA) study cohort. We retrospectively reviewed 60 patients with clinically diagnosed ALGS at Seoul National University Hospital. Forty-three patients with genetically confirmed disease were analyzed. Eight patients (18.6%) underwent liver transplantation (median age: 3.9 years), revealing a lower rate than that in the comparator cohort. The estimated NLS percentages at 5, 10, and 18 years were 86.9%, 86.9%, and 76.6%, respectively, exceeding those in previous reports. The corresponding OS rates were 90.2%, 86.9%, and 86.9%, respectively. The following types of *JAG1* variants were identified in 41 patients (95.3%): frameshift (34.1%), nonsense (26.8%), missense (24.4%), and splice-site (9.8%) variants and in-frame deletions (4.9%). Compared with the reference group, our cohort exhibited a greater frequency of non-protein-truncating variants (missense variants and in-frame deletions; *p* = 0.023) and no structural variants (*p* = 0.043). Two patients (4.7%) carried *NOTCH2* nonsense variants. Mortality was significantly higher among patients with frameshift variants compared with patients with non-frameshift variants (4 of 5 deaths;* p* = 0.035).

*Conclusion*: Compared with the GALA cohort, Korean patients with ALGS exhibited more favorable long-term NLS and a higher proportion of non-protein-truncating *JAG1* variants, alongside the absence of structural variants. These findings suggest potential genetic influences and highlight the need for multicenter validation.

**Table:** 

**What is Known:** *• The Global ALagille Alliance (GALA) study reports native liver survival (NLS) rates of 66.8%, 54.4%, and 40.3% at 5, 10, and 18 years, respectively, in patients with Alagille syndrome.* *• NLS rates are higher among Asians, with unclear genotype–phenotype correlations.*
**What is New:** *• Compared to the GALA study cohort, the Korean cohort demonstrates superior NLS rates of 86.9%, 86.9%, and 76.6% at 5, 10, and 18 years and a higher frequency of non-truncating JAG1 variants, along with the absence of structural variants.* *• Protein-truncating variants are associated with higher initial gamma-glutamyl transferase levels, while frameshift variants are associated with reduced overall survival.*

**Graphical Abstract:**

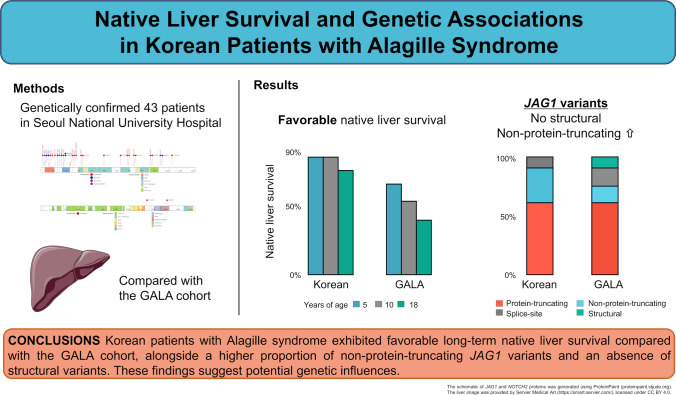

**Supplementary Information:**

The online version contains supplementary material available at 10.1007/s00431-026-06917-3.

## Introduction

Alagille syndrome (ALGS, OMIM #118450 (ALGS1), #610205 (ALGS2)) is a multisystemic autosomal dominant disorder and the most frequent inherited cause of neonatal cholestasis, occurring with an overall incidence of approximately 1 in 30,000 live births [1, 2]. ALGS is characterized by neonatal cholestatic liver disease, cardiac disease, and distinctive facial features, with additional abnormalities involving the spine, blood vessels, kidneys, and eyes [3].

Previous research focused on ALGS patients has revealed pathogenic variants primarily in the Notch signaling ligand *Jagged1* (*JAG1*), with less frequent variants observed in the Notch receptor gene *NOTCH2* [4]. Most pathogenic variants in *JAG1* are protein-truncating variants, such as frameshift, nonsense, and splice-site variants, with missense variants accounting for approximately 15% of cases. In contrast, the *NOTCH2* variants linked to ALGS are mostly missense variants. Whole-gene deletions and intragenic variants in *JAG1* result in similar clinical features, consistent with a haploinsufficiency mechanism. Previous attempts to correlate variant location with clinical symptoms have not been successful [2, 4, 5].

The Global ALagille Alliance (GALA) Study Group, established in 2018, currently encompasses 67 pediatric centers across 29 countries. Its principal aim is to clarify the natural history of liver disease in a contemporary, multinational cohort of children diagnosed with ALGS. The GALA study revealed significant regional disparities in native liver survival (NLS) rates, with the Asian cohort exhibiting a 10-year NLS rate of 73.6% compared with 54.4% in the overall cohort [6]. However, the study did not explore or explain the reasons underlying these observed differences across regions.

Despite advances in our understanding of the genetic basis of ALGS, the influence of specific variant types and their contributions to clinical heterogeneity remain incompletely defined, particularly across diverse populations. Moreover, regional differences in disease course and outcomes have been observed; however, the causes for these discrepancies remain unknown.

This study aims to comprehensively characterize the genetic landscape of ALGS in a Korean cohort completely independent of the GALA cohort, assess its impact on long-term NLS and overall survival (OS), and compare these findings with those in the GALA cohort. By elucidating genetic variants linked to clinical outcomes, we seek to improve prognostic accuracy in ALGS.

## Materials and methods

### Study design and population

This study retrospectively reviewed the medical records of 60 patients who were clinically diagnosed with ALGS and managed at Seoul National University Hospital (SNUH) between January 2000 and July 2025. Genetic testing was performed in 54 patients; six underwent chromosomal microarray analysis without sufficient resolution for definitive variant classification, four had negative results, and one had incomplete documentation limited to the initial visit. After exclusions, 43 patients from 39 unrelated families with genetically confirmed ALGS constituted the analytical cohort. Among these, genetic variants from 17 patients have been previously documented at our center [7, 8]. Importantly, the cohort is entirely independent of the GALA study cohort, with no patient overlap. Genetic confirmation was attained by either direct Sanger sequencing or a targeted gene panel for cholestasis. Comprehensive data, including familial history, clinical manifestations, laboratory findings, imaging modalities (including ultrasound, echocardiogram, and magnetic resonance imaging), histopathological evaluations, genetic analyses, and both medical and surgical therapeutic interventions, were meticulously collected. Medical data were extracted as pseudonymized information from the SNUH web database using CDW SUPREME 2.0 software.

ALGS diagnosis was established when a patient had at least three characteristic disease features or had one disease feature combined with either a family history of this diagnosis in a first-degree relative or a confirmed pathogenic or likely pathogenic variant in *JAG1* or *NOTCH2* [2]. Hepatic involvement or cholestasis at any age was defined by the presence of one or more of the following parameters: conjugated bilirubin concentrations greater than 1.0 mg/dL, gamma-glutamyl transferase (GGT) levels elevated to more than three times the age-appropriate upper limit, elevated liver aminotransferases, histological abnormalities, a history of pruritus, histopathological abnormalities consistent with cholestasis, or a prior history of hepatobiliary surgical interventions. OS was defined as the time from birth to either death or the most recent clinical follow-up, whichever occurred first. NLS was defined as the time from birth to either liver transplantation or the most recent clinical follow-up, whichever occurred first. Only patients who presented with hepatic involvement or cholestasis were included in the NLS analysis.

### Genetic testing

Initial genetic testing involved Sanger sequencing of the *JAG1* gene along with multiplex ligation-dependent probe amplification (MLPA). If the clinical differentiation or diagnosis of cholestasis remained uncertain, a targeted gene panel for cholestasis was subsequently performed. Library preparation, including the construction of precapture libraries (Illumina, Inc., San Diego, CA, USA) and the capture process (Agilent Technologies, Santa Clara, CA, USA), was performed following the manufacturer’s standard protocols. The enriched libraries were subsequently sequenced using the MiSeqDx platform (Illumina, Inc., San Diego, CA, USA). Raw sequencing data were processed and analyzed with NextGENe software (SoftGenetics, State College, PA, USA), and variant annotation was conducted using ANNOVAR to interpret the identified genetic variants [7].

Variants were filtered using the Genome Aggregation Database (gnomAD, gnomad.broadinstitute.org) [9] and the Korean Variant Archive (KOVA, kobic.re.kr/kova/) to exclude common population variants, whereas previously reported pathogenicity was assessed through cross-referencing with the Varsome [10] and ClinVar [11] databases. All variants were interpreted according to the 2015 guidelines of the American College of Medical Genetics and Genomics and the Association for Molecular Pathology (ACMG/AMP) [12] and were further evaluated using recent gene-specific recommendations for *JAG1* and *NOTCH2* variants associated with ALGS [2]. The detected variants were classified as protein-truncating (frameshift or nonsense), non-protein-truncating (missense or in-frame deletion), or splice-site variants. Structural variants were defined as full gene deletions, single- or multiexon deletions, full-gene or multiexon duplications, or translocations.

To evaluate their potential functional impacts, the frameshift, splice-site, and nonsense variants were further assessed using in silico prediction tools, including Combined Annotation Dependent Depletion (CADD, version 1.7, cadd.gs.washington.edu) [13], MutPred (mutpred.mutdb.org) [14], and SpliceAI (spliceailookup.broadinstitute.org) [15]. Missense variants were additionally evaluated using Polymorphism Phenotyping v2 (PolyPhen-2, genetics.bwh.harvard.edu/pph2/) [16], Sorting Intolerant From Tolerant (SIFT 4G, sift.bii.a-star.edu.sg/sift4g/) [17], CADD (version 1.7), and MutPred. Subgroup analyses were conducted on the basis of variant type (frameshift versus non-frameshift; protein-truncating versus non-protein-truncating; protein-truncating versus missense). Owing to the low frequency of *NOTCH2* variants (n = 2), analyses stratified by gene locus were not performed.

### Statistical analyses

The survival probabilities for NLS and OS were estimated by univariate Kaplan‒Meier analysis, with subgroup differences assessed using the log-rank test. Variant distributions were compared with those of the GALA cohort [2] using Fisher’s exact test. Initial laboratory values in cholestatic patients were compared between protein-truncating and non-protein-truncating variants by performing a Mann‒Whitney U test. All the statistical analyses were performed using R software (version 4.5.1; R Foundation for Statistical Computing, Vienna, Austria), and statistical significance was defined as two-sided *p* < 0.05.

## Results

### Patient characteristics

Among the 43 patients with genetically confirmed ALGS (Table 1), 55.8% were male, with a median age at symptom onset of 2.1 months (interquartile range, IQR, 0.9–4.1). Hepatic involvement or cholestasis was observed in 95.3% of patients, and bile duct paucity was documented in 70.4% of patients who underwent liver biopsy. Cardiac anomalies and characteristic facial features were each present in 90.7% of the patients. Renal anomalies were identified in 51.2% of the patients, skeletal abnormalities in 46.5%, ocular features in 32.5%, and vascular anomalies in 33.3%. During the follow-up period, eight patients (18.6%) underwent liver transplantation because of complications from persistent cholestasis at a median age of 3.9 years, and five patients (11.6%) died.

**Table 1:** Patient characteristics

	All (*n* = 43)
Male, % (*n*)	55.8% (*n* = 24)
Age at symptom onset, months, median (IQR)	2.1 (0.9–4.1)
**Clinical features, % (*n*)**	
Hepatic involvement, any	95.3% (*n* = 41)
Bile duct paucity	70.4% (*n* = 19/27)
Echo-confirmed cardiac anomaly, any	90.7% (*n* = 39)
Characteristic facial features	90.7% (*n* = 39)
Ocular features	32.5% (*n* = 13/40)
Skeletal features	46.5% (*n* = 20)
Renal anomaly, any	51.2% (*n* = 22)
Vascular anomaly, any	33.3% (*n* = 9/27)
**Clinical outcomes, % (*n*)**	
Liver transplantation	18.6% (*n* = 8)
Age at transplant, years, median (IQR)	3.9 (1.4–13.6)
Death	11.6% (*n* = 5)
**Genetic variants, % (*n*)**	
*JAG1*	95.3% (*n* = 41)
*NOTCH2*	4.7% (*n* = 2)

*IQR* Interquartile range, *Echo* echocardiogram

### Initial laboratory findings

Initial laboratory findings for ALGS patients with *JAG1* variants presenting with hepatic involvement or cholestasis are summarized in Table 2. Patients harboring protein-truncating variants tended to exhibit higher median levels of total bilirubin, conjugated bilirubin, aspartate aminotransferase (AST), alanine aminotransferase (ALT), and GGT than did patients with non-protein-truncating variants. Among these parameters, only GGT levels exhibited a statistically significant difference (*p* = 0.037). Missense variants predominated among non-protein-truncating variants (83.3%). Direct comparison revealed significantly lower initial GGT levels in missense versus protein-truncating variant carriers (*p* = 0.035, Table 2).

**Table 2:** Initial laboratory findings in patients with *JAG1* variant-associated ALGS who presented with hepatic involvement or cholestasis, reported as median (IQR)

	All (*n* = 41)	Protein-truncating (*n* = 25)	Non-protein-truncating (*n* = 12)	*p* value^a^	Missense (*n* = 10)	*p* value^b^
Total bilirubin, mg/dL	3.5 (1.2–7.7)	5.0 (1.1–7.8)	3.9 (2.2–9.4)	0.670	3.9 (1.9–7.7)	0.872
Conjugated bilirubin, mg/dL	3.9 (0.9–5.7)	4.8 (1.2–5.7)	3.1 (1.3–6.0)	0.663	3.1 (2.2–5.8)	0.577
AST, IU/L	151.0 (96.5–238.0)	173.5 (121.3–247.3)	97.0 (79.0–197.5)	0.183	97.0 (79.0–124.0)	0.140
ALT, IU/L	139.0 (112.5–259.5)	139.0 (115.0–255.8)	117.0 (51.0–211.0)	0.278	117.0 (59.0–180.0)	0.249
GGT, IU/L	483.0 (185.0–700.0)	556.5 (288.3–866.8)	185.0 (90.5–555.0)	0.037*	185.0 (92.0–483.0)	0.035*
Cholesterol, mg/dL	224.0 (188.0–345.0)	224.0 (191.0–325.5)	207.0 (138.0–336.0)	0.481	207.0 (164.0–334.0)	0.416
Platelet count, 10^9^/L	398.5 (315.3–537.5)	444.0 (331.5–573.0)	525.0 (315.5–589.5)	0.898	530.0 (316.0–639.0)	0.630

*IQR* Interquartile range, *ALT* Alanine aminotransferase, *AST* Aspartate aminotransferase, *GGT* Gamma‐glutamyl transferase

Protein-truncating: frameshift or nonsense

Non-protein-truncating: missense or in-frame deletion

^a^ Protein-truncating versus non-protein-truncating

^b^ Missense versus protein-truncating

* Statistically significant

### Genetic spectrum

**Fig. 1:**
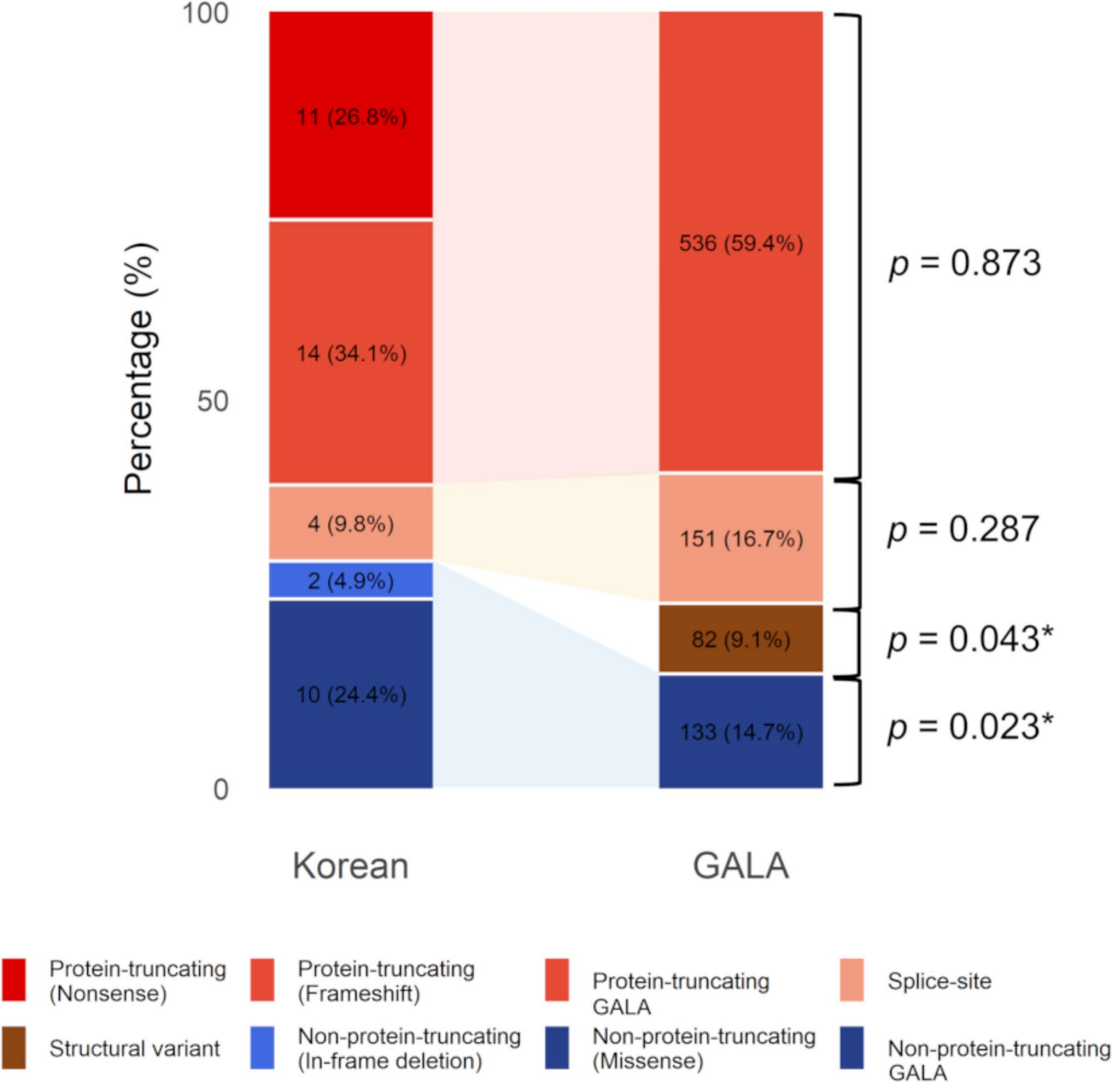
Comparison of *JAG1* variant types with those in the GALA cohort. ^*^Statistically significant

*JAG1* variants, including frameshift (34.1%), nonsense (26.8%), missense (24.4%), splice-site (9.8%), and in-frame deletion (4.9%) variants, were identified in 95.3% of the patients. Compared with the GALA international cohort, the Korean cohort exhibited a significantly higher frequency of non-protein-truncating variants (29.3% versus 14.7%, *p* = 0.023) and a complete absence of structural variants (0% versus 9.1%, *p* = 0.043), whereas the prevalence of protein-truncating and splice-site variants was comparable (Fig. 1). Two patients (4.7%) carried *NOTCH2* nonsense variants.

**Fig. 2:**
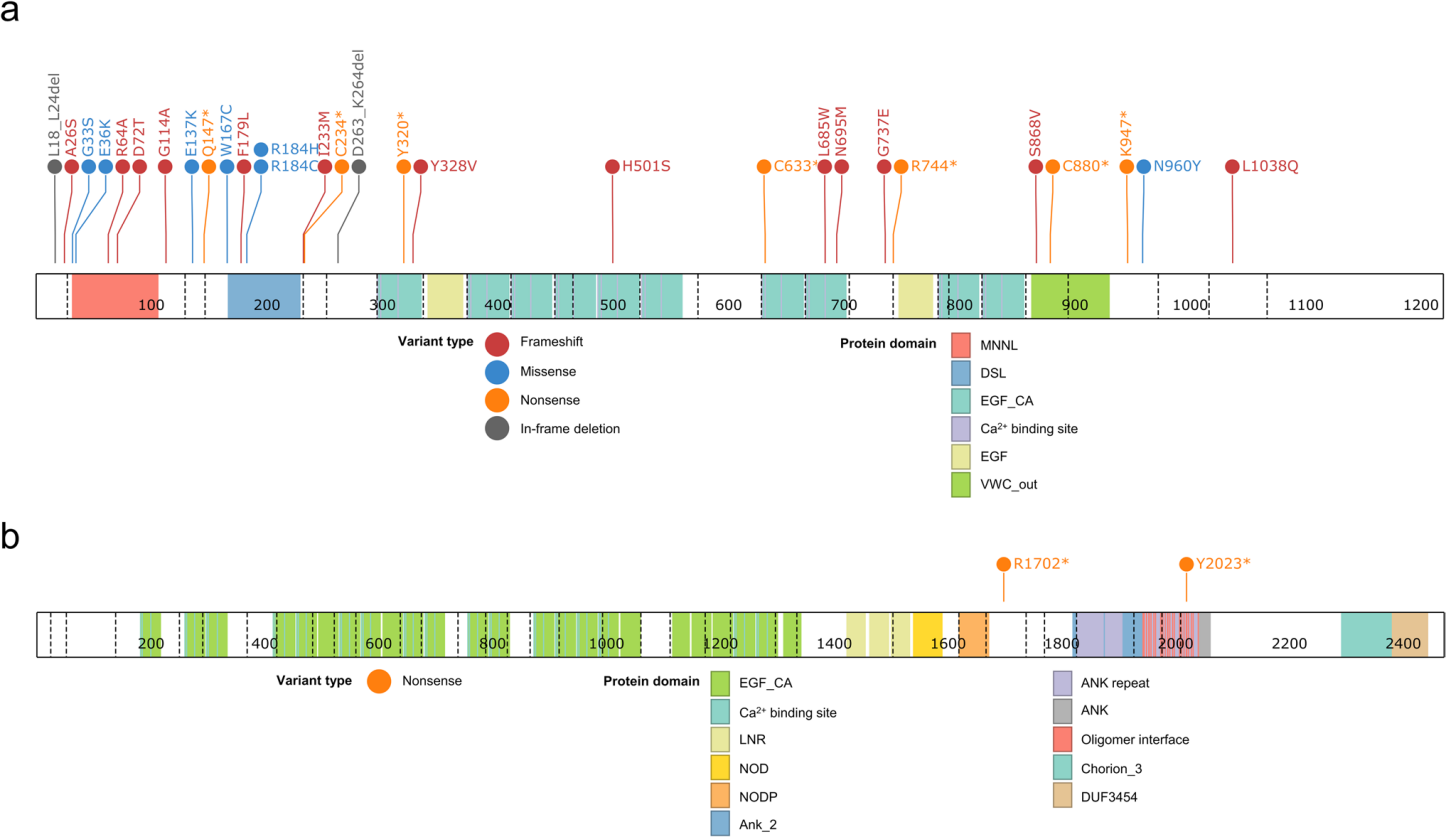
Schematic of *JAG1* and *NOTCH2* proteins in ALGS patients. A total of 33 genetic variants in the *JAG1* gene and 2 variants in the *NOTCH2* gene were identified in ALGS patients. The figure depicts all identified genetic variants, excluding four splice-site variants in *JAG1*. The image was generated using ProteinPaint (proteinpaint.stjude.org). The classification of genetic variants including full Human Genome Variation Society (HGVS) nomenclature is detailed in Supplementary Table 2. (**a**) *JAG1*. (**b**) *NOTCH2*. Abbreviations: ANK, ankyrin repeats; ANK_2, ankyrin repeats (3 copies); ANK repeat, ANK repeat [structrural motif]; Chorin_3, Chorion family 3; DUF3454, Domain of unknown function (DUF3454); DSL, Delta/Serrate/Lag-2 domain; EGF_CA, Calcium-binding epidermal growth factor-like domain; EGF-like, epidermal growth factor-like repeats; LNR, Lin-12/Notch repeats; MNNL, N terminus of Notch ligand; NOD, NOTCH protein; NODP, NOTCH protein; VWC_out, von Willebrand factor type C domain

On the basis of ACMG/AMP classification guidelines [12] and recent recommendations for *JAG1* and *NOTCH2* variants associated with ALGS [2], the majority of variants were classified as pathogenic or likely pathogenic. One patient harboring a variant of uncertain significance (VUS) was included because of the presence of three characteristic clinical features of ALGS. Figure 2 depicts the schematic representation of *JAG1* and *NOTCH2* proteins with their respective genetic variants. The classification of genetic variants including Human Genome Variation Society (HGVS) nomenclature and their associated clinical outcomes are detailed in Supplementary Tables 1 and 2.

### Survival outcomes

**Fig. 3:**
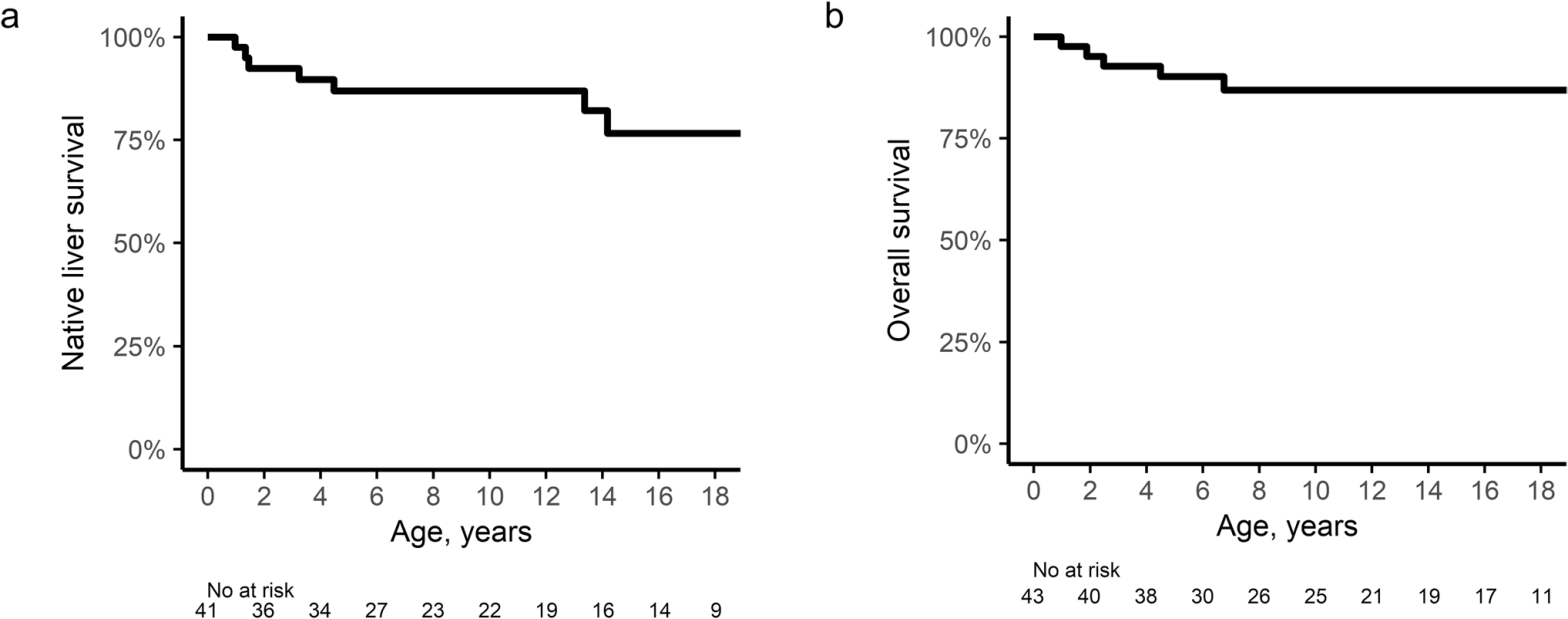
Survival outcomes (**a**) Native liver survival (NLS) rates in ALGS patients with a *JAG1* or *NOTCH2* variant presenting with hepatic involvement or cholestasis (**b**) Overall survival (OS) rates in ALGS patients with a *JAG1* or *NOTCH2* variant

The Kaplan–Meier estimates of NLS were 86.9%, 86.9%, and 76.6% at 5, 10, and 18 years of age, respectively, whereas the OS rates were 90.2%, 86.9%, and 86.9%, respectively, at the same time points (Fig. 3). Survival analyses showed no significant NLS or OS differences between protein-truncating versus non-protein-truncating variants (Supplementary Fig. 1) or protein-truncating versus missense variants.

Among the five deaths observed in this context, four occurred in frameshift carriers, whereas one occurred in a patient with a missense variant. Mortality was significantly greater in patients carrying frameshift variants compared with those with non-frameshift variants (4 out of 5 deaths; *p* = 0.035). The causes of death among the five patients varied and included two cases of acute complications shortly after liver transplantation, gastrointestinal bleeding due to portal vein occlusion, cardiac complications, and trauma-induced epidural hemorrhage.

**Fig. 4:**
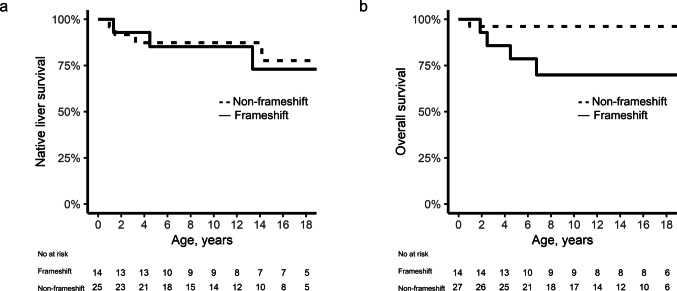
Survival outcomes for frameshift versus non-frameshift variants (**a**) Native liver survival (NLS) rates in ALGS patients with a *JAG1* variant presenting with hepatic involvement or cholestasis (log-rank *p* = 0.577) (**b**) Overall survival (OS) rates in ALGS patients with a *JAG1* variant (log-rank *p* = 0.032)

Given that mortality was significantly associated with patients carrying frameshift variants, we performed a comparison between frameshift and non-frameshift variant groups. Among patients who harbored *JAG1* variants with hepatic involvement or cholestasis at presentation, NLS did not differ significantly between patients with frameshift variants and those with non-frameshift variants (log-rank *p* = 0.577). In contrast, OS was significantly lower in patients with frameshift variants (log-rank *p* = 0.032) (Fig. 4).

## Discussion

This study provides a comprehensive, long-term, genetic and clinical characterization of Korean patients with ALGS, revealing clinical outcomes distinct from those of international cohorts. Compared with the overall GALA cohort, the Korean cohort demonstrated substantially higher NLS [6], with estimates of 86.9%, 86.9%, and 76.6% at 5, 10, and 18 years of age, respectively, compared with 66.8%, 54.4%, and 40.3%, respectively, in the GALA cohort. In the GALA study, patients were additionally stratified into regional subgroups to assess potential geographic disparities. In this analysis, the Asian subgroup of the GALA cohort exhibited higher NLS (77.0%, 73.6%, and 67.9%) compared with that of the overall GALA population. However, these survival estimates were still consistently lower than those observed in the Korean cohort. OS rates were comparable across cohorts, with the overall GALA cohort exhibiting rates of 92.8%, 91.2%, and 88.1% at 5, 10, and 18 years, respectively, paralleling the favorable OS observed in the Korean population. These findings can be attributed to patients with severe liver disease in the GALA cohort undergoing liver transplantation and subsequently achieving favorable posttransplant survival.

The GALA study retrospectively reviewed pediatric ALGS patients who were diagnosed clinically or genetically and who were born between January 1997 and August 2019 [6]; this study shares methodological similarities with our investigation. However, our study included patients with exclusively genetically confirmed ALGS, whereas only approximately 60% of the GALA cohort received genetic confirmation of ALGS. This difference introduces heterogeneity, and direct comparisons should be interpreted cautiously because of potential confounding from diagnostic criteria and patient characterization.

By employing a genetically validated cohort, our study reduces phenotypic heterogeneity and increases the precision of genotype‒phenotype correlations, thereby refining prognostic assessments. Despite the limitations inherent in cross-cohort comparisons, our findings offer novel insights that complement and extend previous research on the clinical spectrum and outcomes of ALGS.

Significantly, this study elucidates factors that contribute to the elevated NLS observed in the Asian subset of the GALA cohort. Genetically, the distribution of *JAG1* variants in our cohort aligns with global trends [2], which predominantly feature frameshift and nonsense variants. However, this distribution differs on the basis of the higher prevalence of non-protein-truncating variants and complete absence of structural variants observed in our study cohort. Notably, a recent study reported that missense variants occur at a higher frequency in Asian populations than in non-Asian populations [5], suggesting that population-specific variant spectra may influence clinical presentation and outcomes.

Although non-protein-truncating variants have been hypothesized to be associated with milder disease, marked phenotypic variability and the absence of definitive genotype‒phenotype correlations in the literature suggest that other genetic modifiers, such as the transcription factor SOX9 and Thrombospondin 2 (THBS2), critically influence disease expression [3, 18]. Our identification of an increased frequency of non-protein-truncating variants associated with favorable hepatic outcomes in this genetically confirmed Korean cohort represents a novel contribution, suggesting that population-specific *JAG1* variant distributions may influence disease severity. In support of these findings, initial laboratory data for ALGS patients with *JAG1* variants presenting with hepatic involvement or cholestasis revealed that GGT levels differed significantly between protein-truncating and non-protein-truncating variant carriers. Notably, missense variants predominated among non-protein-truncating variants and were associated with significantly lower initial GGT levels compared to those in patients with protein-truncating variants. This finding suggests that non-protein-truncating variants, particularly missense variants, may be associated with a milder liver phenotype in ALGS patients. However, population-specific genetic background effects independent of *JAG1* variants warrant consideration in interpreting these findings. Moreover, NLS did not differ significantly between patients with protein-truncating and non-protein-truncating variants, consistent with findings from the GALA study [2]. In the Taiwanese cohort, patients who achieved jaundice-free status versus those with progressive disease showed no clear genotype‒phenotype correlation [19]. Accordingly, this hypothesis should be interpreted cautiously given the limited number of missense variants (*n* = 10) in our cohort. Further studies involving larger, ethnically diverse cohorts and functional analyses are essential to clarify the mechanisms that underlie phenotypic heterogeneity and to facilitate precision prognostics and personalized management.

Survival analysis revealed a variant-specific effect on OS, with frameshift variants significantly associated with reduced OS, whereas NLS was not influenced by variant type. Among the three patients who died after liver transplantation, two carried frameshift variants, and one harbored a missense variant (*JAG1* c.551G > A, p.(Arg184His)). This missense variant was previously functionally analyzed by Tada et al. [20], who demonstrated retained Notch-binding activity comparable to that of the wild-type protein but markedly impaired *trans*-activation and *cis*-inhibition functions. Compared with the wild-type protein, the variant protein predominantly localized to the endoplasmic reticulum (ER), indicating improper folding, and showed increased binding to the ER chaperones calnexin and calreticulin [20]. This variant was also identified in an ALGS patient with hepatocellular carcinoma [21], thus distinguishing it from other missense variants and potentially explaining the associated mortality.

The causes of death among the five deceased patients were diverse and included gastrointestinal bleeding secondary to portal vein occlusion, cardiac complications, and trauma-related epidural hemorrhage. This heterogeneous mortality profile highlights the multifactorial risks faced by ALGS patients and the need for comprehensive multidisciplinary care.

This study is limited by its modest sample size, retrospective single-center design, and relatively homogeneous ethnicity, which may restrict generalizability, although such homogeneity also allowed the detailed characterization of cohort-specific genetic profiles. While our higher non-truncating *JAG1* variant frequency associates with superior NLS, several factors necessitate cautious interpretation. Cross-cohort comparisons cannot disentangle direct variant effects from the effects of population-specific genetic background factors or differences in clinical management. Limited event numbers reduce statistical power for survival subgroup analyses. Validation by reference to larger, multicenter, and ethnically diverse cohorts is warranted to confirm these observations and to elucidate the mechanistic roles of non-protein-truncating variants in disease modulation. Future research integrating functional assays and comprehensive genomic analyses will be critical to improve our understanding of genotype–phenotype relationships and to optimize individualized therapeutic strategies in ALGS.

In conclusion, this study highlights the contributions of population-specific *JAG1* genetic architecture and variant-level heterogeneity to ALGS clinical variability. Compared with the international cohort, the Korean cohort exhibited favorable NLS, and the introduction of ileal bile acid transporter inhibitors may further improve NLS by postponing the need for liver transplantation. Importantly, frameshift variants were associated with poor OS, underscoring the prognostic utility of detailed genetic classification.

## Supplementary Information

Below is the link to the electronic supplementary material.

ESM 1 (PDF 481 KB)

## Data Availability

The relevant data associated with this study are included within the article and Supporting Information.

## Abbreviations

ALGS: Alagille syndrome
GALA: Global ALagille Alliance
GGT: Gamma-glutamyl transferase
*JAG1*: *Jagged1*
NLS: Native liver survival
OS: Overall survival

## References

[1] Karpen SJ, Kamath BM, Alexander JJ, Ichetovkin I, Rosenthal P, Sokol RJ, Dunn S, Thompson RJ, Heubi JE (2021) Use of a comprehensive 66-gene cholestasis sequencing panel in 2171 cholestatic infants, children, and young adults. J Pediatr Gastroenterol Nutr 72(5):654–660. 10.1097/MPG.0000000000003094 33720099 10.1097/MPG.0000000000003094

[2] Vandriel SM, Li L, She H et al (2025) Phenotypic divergence of JAG1 ‐ and NOTCH2 ‐associated Alagille syndrome & disease‐specific NOTCH2 variant classification guidelines. Liver Int 45(9):e70251. 10.1111/liv.70251 40742203 10.1111/liv.70251 PMC12312628

[3] Kohut TJ, Gilbert MA, Loomes KM (2021) Alagille syndrome: a focused review on clinical features, genetics, and treatment. Semin Liver Dis 41(04):525–537. 10.1055/s-0041-1730951 34215014 10.1055/s-0041-1730951

[4] Gilbert MA, Bauer RC, Rajagopalan R et al (2019) Alagille syndrome mutation update: comprehensive overview of JAG1 and NOTCH2 mutation frequencies and insight into missense variant classification. Hum Mutat 40(12):2197–2220. 10.1002/humu.23879 31343788 10.1002/humu.23879 PMC6899717

[5] Sharma P, Abbey D (2025) Alagille syndrome: unraveling the complexities of genotype-phenotype relationships and exploring avenues for improved diagnosis and treatment. Cell Biol Int 49(5):435–471. 10.1002/cbin.70009 40042123 10.1002/cbin.70009

[6] Vandriel SM, Li L, She H et al (2023) Natural history of liver disease in a large international cohort of children with Alagille syndrome: results from the GALA study. Hepatology 77(2):512–529. 10.1002/hep.32761 36036223 10.1002/hep.32761 PMC9869940

[7] Hahn JW, Lee H, Shin M, Seong MW, Moon JS, Ko JS (2024) Diagnostic algorithm for neonatal intrahepatic cholestasis integrating single-gene testing and next-generation sequencing in East Asia. J Gastroenterol Hepatol 39(5):964–974. 10.1111/jgh.16505 38323732 10.1111/jgh.16505

[8] Moon JS (2007) JAG1 Mutation Analysis and the Whole Genomic Screening using Microarray-Based Comparative Genomic Hybridization in Korean Children with Alagille Syndrome. Dissertation, Seoul National University

[9] Karczewski KJ, Francioli LC, Tiao G et al (2020) The mutational constraint spectrum quantified from variation in 141,456 humans. Nature 581(7809):434–443. 10.1038/s41586-020-2308-7 32461654 10.1038/s41586-020-2308-7 PMC7334197

[10] Kopanos C, Tsiolkas V, Kouris A, Chapple CE, Albarca Aguilera M, Meyer R, Massouras A (2019) VarSome: the human genomic variant search engine. Bioinformatics 35(11):1978–1980. 10.1093/bioinformatics/bty897 30376034 10.1093/bioinformatics/bty897 PMC6546127

[11] Landrum MJ, Lee JM, Riley GR, Jang W, Rubinstein WS, Church DM, Maglott DR (2014) ClinVar: public archive of relationships among sequence variation and human phenotype. Nucleic Acids Res 42(Database issue):D980-985. 10.1093/nar/gkt1113 24234437 10.1093/nar/gkt1113 PMC3965032

[12] Richards S, Aziz N, Bale S et al (2015) Standards and guidelines for the interpretation of sequence variants: a joint consensus recommendation of the American College of Medical Genetics and Genomics and the Association for Molecular Pathology. Genet Med 17(5):405–424. 10.1038/gim.2015.30 25741868 10.1038/gim.2015.30 PMC4544753

[13] Schubach M, Maass T, Nazaretyan L, Röner S, Kircher M (2024) CADD v1.7: using protein language models, regulatory CNNs and other nucleotide-level scores to improve genome-wide variant predictions. Nucleic Acids Res 52(D1):D1143–D1154. 10.1093/nar/gkad989 38183205 10.1093/nar/gkad989 PMC10767851

[14] Pejaver V, Urresti J, Lugo-Martinez J et al (2020) Inferring the molecular and phenotypic impact of amino acid variants with MutPred2. Nat Commun 11(1):5918. 10.1038/s41467-020-19669-x 33219223 10.1038/s41467-020-19669-x PMC7680112

[15] Jaganathan K, Kyriazopoulou Panagiotopoulou S, McRae JF et al (2019) Predicting Splicing from Primary Sequence with Deep Learning. Cell 176(3):535-548.e24. 10.1016/j.cell.2018.12.015 30661751 10.1016/j.cell.2018.12.015

[16] Adzhubei IA, Schmidt S, Peshkin L, Ramensky VE, Gerasimova A, Bork P, Kondrashov AS, Sunyaev SR (2010) A method and server for predicting damaging missense mutations. Nat Methods 7(4):248–249. 10.1038/nmeth0410-248 20354512 10.1038/nmeth0410-248 PMC2855889

[17] Ng PC, Henikoff S (2001) Predicting deleterious amino acid substitutions. Genome Res 11(5):863–874. 10.1101/gr.176601 11337480 10.1101/gr.176601 PMC311071

[18] Halma J, Lin HC (2023) Alagille syndrome: understanding the genotype-phenotype relationship and its potential therapeutic impact. Expert Rev Gastroenterol Hepatol 17(9):883–892. 10.1080/17474124.2023.2255518 37668532 10.1080/17474124.2023.2255518

[19] Chiang C, Jeng Y, Ho M et al (2022) Different clinical and genetic features of Alagille patients with progressive disease versus a jaundice‐free course. JGH Open 6(12):839–845. 10.1002/jgh3.12830 36514505 10.1002/jgh3.12830 PMC9730729

[20] Tada M, Itoh S, Ishii-Watabe A, Suzuki T, Kawasaki N (2012) Functional analysis of the Notch ligand Jagged1 missense mutant proteins underlying Alagille syndrome. FEBS J 279(12):2096–2107. 10.1111/j.1742-4658.2012.08595.x 22487239 10.1111/j.1742-4658.2012.08595.x

[21] Valamparampil JJ, Shanmugam N, Vij M, Reddy MS, Rela M (2020) Hepatocellular carcinoma in paediatric patients with Alagille syndrome: case series and review of literature. J Gastrointest Cancer 51(3):1047–1052. 10.1007/s12029-020-00391-2 32180165 10.1007/s12029-020-00391-2

