# Supplementary material for: Native liver survival and genetic associations in Korean patients with Alagille syndrome
Source: Eur J Pediatr. 2026 Apr 10;185(5):252. doi: 10.1007/s00431-026-06917-3 (PMC13068675; doi:10.1007/s00431-026-06917-3)
Supplement: Supplementary file 1 — (PDF 481 KB) [file 431_2026_6917_MOESM1_ESM.pdf]

Supplementary Table 1 Phenotypes and clinical outcomes

Patient no.	Sex	Gene	Phenotypes	Cardiac anomalies	Outcome
1	F	*JAG1*	F, H, L, R	PS, ASD	LT
2	F	*JAG1*	F, H, L, S	PS	LT, D
3	M	*JAG1*	E, F, FDR, H, S	PS, ASD	-
4	F	*JAG1*	F, FDR, H, L, S	TOF	-
5	F	*JAG1*	F, H, L, S	PS	-
6	F	*JAG1*	F, H, L, R, V	PS, AS	LT
7	F	*JAG1*	F, H, L, R, S	PA, VSD, AS	D
8	M	*JAG1*	F, H, L, R	PS	-
9	M	*JAG1*	E, F, H, L, R, S	PS	-
10	M	*JAG1*	F, H, L, S	PS, ASD, PDA	-
11	M	*JAG1*	F, H, L	PS	-
12	F	*JAG1*	F, H, L, R	PS, ASD	-
13	F	*JAG1*	F, H, L, R	PS, PAPVR	-
14	M	*JAG1*	H, L, S	PS, ASD	-

15	M	*JAG1*	F, H, L, R	PS	-
16	F	*JAG1*	E, F, H, L, S	PS, ASD	LT, D
17	F	*JAG1*	F, H, L, R	IAA, VSD, PDA, PS, AS	LT, D
18	M	*JAG1*	F, FDR, H, L	PS, ASD, PDA	-
19	F	*JAG1*	F, FDR, L	-	-
20	M	*JAG1*	E, H, L, S	DORV, PA, VSD, PDA	-
21	M	*JAG1*	H, L, R	PS, ASD	-
22	M	*JAG1*	F, FDR, H, L	PS	-
23	F	*JAG1*	F, H, L, R, S	PS	LT
24	M	*JAG1*	F, H, L, R, S, V	PS	-
25	F	*JAG1*	E, F, H, L, S, V	VSD, PS, ASD, PDA	-
26	F	*JAG1*	E, F, H, R, S, V	VSD	-
27	M	*JAG1*	F, L, R, V	-	-
28	M	*JAG1*	F, H, L	PS	-
29	M	*JAG1*	E, F, H, L, R, V	PS	-
30	M	*JAG1*	E, F, FDR, H, L, R, S	PS	-
31	F	*JAG1*	E, F, FDR, H, L, V	ASD	-

32	M	*JAG1*	F, FDR, H, L, R	PS, ASD	-
33	F	*JAG1*	E, F, FDR, H, L, R	PS	-
34	F	*JAG1*	E, F, H, L, S	VSD	-
35	M	*JAG1*	F, H, L, S	PS	-
36	M	*JAG1*	E, F, FDR, H, L, R, V	PS	LT
37	F	*JAG1*	E, F, FDR, H, L	PS	-
38	M	*JAG1*	F, H, L, R, S	PS, ASD	LT
39	F	*JAG1*	F, L	-	-
40	M	*JAG1*	F, L, S	-	-
41	M	*JAG1*	F, FDR, H, L, R, S, V	PS	D
42	M	*NOTCH2*	F, H, L, R	ASD	-
43	M	*NOTCH2*	FDR, H, L	PDA	-

Abbreviations: E, eye; F, face; FDR, first-degree relative history; H, heart; L, liver; R, renal; S, skeletal; V,vascular; ASD, atrial septal defect; AS, aortic stenosis; DORV, double outlet right ventricle; IAA, interrupted aortic arch; PAPVR, partial anomalous pulmonary venous return; PA, pulmonary atresia; PDA, patent ductus arteriosus; PS, pulmonary stenosis; TOF, tetralogy of fallot; VSD, ventricular septal defect; LT, liver transplantation; D, death

Supplementary Table 2 Classification of genetic variants

Patient no.	Sex	Gene	Exon/ intron	Coding effect	Nucleotide change	Predicted amino acid change	ACMG evidence	ACMG classification
1	F	*JAG1*	1	In-frame deletion	c.53_73del	p.(Leu18_Leu24del)	PVS1, PS2, PM2	P
2	F	*JAG1*	1	Frameshift	c.74dup	p.(Ala26SerfsTer47)	PVS1, PM2	LP
3	M	*JAG1*	2	Missense	c.97G>A	p.(Gly33Ser)	PM1, PM2, PM5, PP3	LP
4	F	*JAG1*	2	Missense	c.97G>A	p.(Gly33Ser)	PM1, PM2, PM5, PP3	LP
5	F	*JAG1*	2	Missense	c.106G>A	p.(Glu36Lys)	PS3[a], PM1, PM2, PM5, PP3	P
6	F	*JAG1*	2	Frameshift	c.190del	p.(Arg64AlafsTer97)	PVS1, PM2	LP
7	F	*JAG1*	2	Frameshift	c.213_214delTGinsC	p.(Asp72ThrfsTer89)	PVS1, PM2	LP
8	M	*JAG1*	2	Frameshift	c.341del	p.(Gly114AlafsTer47)	PVS1, PS2, PM2	P
9	M	*JAG1*	3	Missense	c.409G>A	p.(Glu137Lys)	PS2, PS3[a], PM1, PM2, PP3	P
10	M	*JAG1*	3	Nonsense	c.439C>T	p.(Gln147Ter)	PVS1, PM2	LP
11	M	*JAG1*	4	Missense	c.501G>T	p.(Trp167Cys)	PS3[a], PM1, PM2, PP3	LP
12	F	*JAG1*	4	Frameshift	c.537del	p.(Phe179LeufsTer7)	PVS1, PS2	LP
13	F	*JAG1*	4	Missense	c.550C>T	p.(Arg184Cys)	PS4, PM1, PM2, PM5, PP3	P
14	M	*JAG1*	4	Missense	c.550C>T	p.(Arg184Cys)	PS2, PS4, PM1, PM2, PM5, PP3	P
15	M	*JAG1*	4	Missense	c.551G>A	p.(Arg184His)	PS3[b], PS4, PM1, PM2, PM5, PP3	P

16	F	*JAG1*	4	Missense	c.551G>A	p.(Arg184His)	PS3[b], PS4, PM1, PM2, PM5, PP3	P
17	F	*JAG1*	5	Frameshift	c.699_700del	p.(Ile233MetfsTer8)	PVS1, PM2	LP
18	M	*JAG1*	5	Nonsense	c.702C>A	p.(Cys234Ter)	PVS1, PM2	LP
19	F	*JAG1*	5	Nonsense	c.702C>A	p.(Cys234Ter)	PVS1, PM2	LP
20	M	*JAG1*	6	In-frame deletion	c.789_791del	p.(Asp263_Lys264delinsGlu)	PVS1, PM2	LP
21	M	*JAG1*	6	Splice site	c.886+2_886+14del	p.?	PVS1, PM2	LP
22	M	*JAG1*	intron6	Splice site	c.887-2A>G	p.?	PVS1, PM2	LP
23	F	*JAG1*	7	Nonsense	c.960T>G	p.(Tyr320Ter)	PVS1, PS2, PM2	P
24	M	*JAG1*	7	Frameshift	c.981dup	p.(Tyr328ValfsTer7)	PVS1, PM2	LP
25	F	*JAG1*	12	Frameshift	c.1499dup	p.(His501SerfsTer5)	PVS1, PM2	LP
26	F	*JAG1*	15	Nonsense	c.1899_1900del	p.(Cys633Ter)	PVS1, PM2	LP
27	M	*JAG1*	intron15	Splice site	c.2000-2A>G	p.?	PVS1, PM2	LP
28	M	*JAG1*	16	Frameshift	c.2053del	p.(Leu685TrpfsTer58)	PVS1, PM2	LP
29	M	*JAG1*	16	Frameshift	c.2084del	p.(Asn695MetfsTer48)	PVS1, PM2	LP
30	M	*JAG1*	17	Frameshift	c.2210del	p.(Gly737GlufsTer6)	PVS1, PM2	LP
31	F	*JAG1*	18	Nonsense	c.2230C>T	p.(Arg744Ter)	PVS1, PM2	LP
32	M	*JAG1*	18	Nonsense	c.2230C>T	p.(Arg744Ter)	PVS1, PM2	LP
33	F	*JAG1*	18	Nonsense	c.2230C>T	p.(Arg744Ter)	PVS1, PM2	LP
34	F	*JAG1*	18	Nonsense	c.2230C>T	p.(Arg744Ter)	PVS1, PM2	LP

35	M	*JAG1*	intron21	Splice site	c.2572+1G>A	p.?	PVS1, PM2	LP
36	M	*JAG1*	22	Frameshift	c.2601del	p.(Ser868ValfsTer2)	PVS1, PM2	LP
37	F	*JAG1*	22	Frameshift	c.2601del	p.(Ser868ValfsTer2)	PVS1, PM2	LP
38	M	*JAG1*	22	Nonsense	c.2639_2640del	p.(Cys880Ter)	PVS1, PM2	LP
39	F	*JAG1*	23	Nonsense	c.2839A>T	p.(Lys947Ter)	PVS1, PM2	LP
40	M	*JAG1*	23	Missense	c.2878A>T	p.(Asn960Tyr)	PM1, PM2, PP3	VUS
41	M	*JAG1*	25	Frameshift	c.3113_3117del	p.(Leu1038GlnfsTer2)	PVS1, PM2	LP
42	M	*NOTCH2*	28	Nonsense	c.5104C>T	p.(Arg1702Ter)	PVS1, PM2	LP
43	M	*NOTCH2*	34	Nonsense	c.6069T>G	p.(Tyr2023Ter)	PVS1, PM2	LP

Abbreviations: PVS1, null variant in a gene where loss of function is a known disease mechanism; PS2, *de novo*; PS3, well-established *in vitro* or *in vivo* functional studies supporting a damaging effect on the gene or gene product; PM1, located in a mutational hot spot and/or critical and well-established functional domain; PM2, absent from controls (gnomAD); PM5, novel missense change at an amino acid residue where a different missense change determined to be pathogenic has been seen before; PP3, multiple lines of computational evidence support a deleterious effect on the gene or gene product; P, pathogenic; LP, likely pathogenic; VUS, variant of uncertain significance

[a] Gilbert MA, Keefer-Jacques E, Jadhav T, et al (2024) Functional characterization of 2,832 JAG1 variants supports reclassification for Alagille syndrome and improves guidance for clinical variant interpretation. Am J Hum Genet 111(8):1656–1672. https://doi.org/10.1016/j.ajhg.2024.06.011

[b] Tada M, Itoh S, Ishii-Watabe A, Suzuki T, Kawasaki N (2012) Functional analysis of the Notch ligand Jagged1 missense mutant proteins underlying Alagille syndrome. FEBS J 279(12):2096–2107. https://doi.org/10.1111/j.1742-4658.2012.08595.x

Supplementary Fig. 1 Survival outcomes for protein-truncating versus non-protein-truncating variants

(a) Native liver survival (NLS) rates in ALGS patients with a *JAG1* variant presenting with hepatic involvement or cholestasis (log-rank $p = 0.989$)

(b) Overall survival (OS) rates in ALGS patients with a *JAG1* variant (log-rank $p = 0.586$)

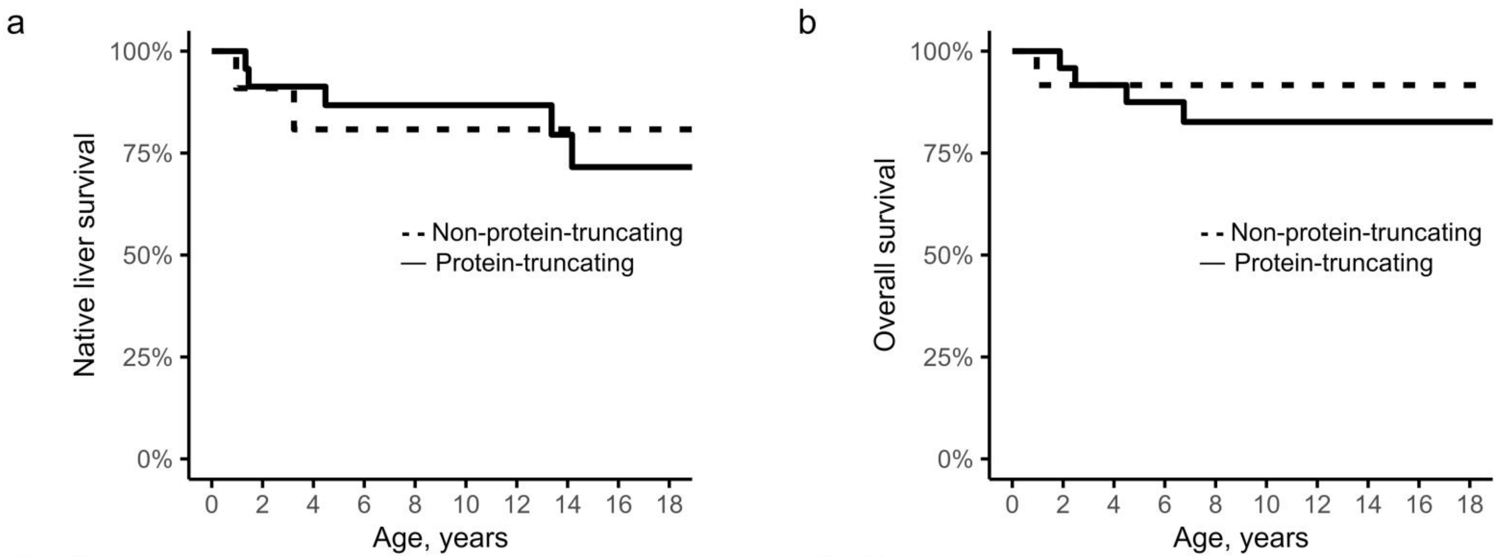

No at risk (a)										
Protein-truncating	24	21	21	17	15	15	13	11	10	7
Non-protein-truncating	11	11	9	7	7	6	5	4	3	2

No at risk (b)										
Protein-truncating	25	24	23	19	17	17	15	14	13	9
Non-protein-truncating	12	12	11	8	8	7	5	4	3	2
